# Abrogation of the radiation-induced G2 checkpoint by the staurosporine derivative UCN-01 is associated with radiosensitisation in a subset of colorectal tumour cell lines

**DOI:** 10.1038/sj.bjc.6600492

**Published:** 2002-08-01

**Authors:** L C Playle, D J Hicks, D Qualtrough, C Paraskeva

**Affiliations:** Cancer Research UK Colorectal Tumour Biology Research Group, Department of Pathology and Microbiology, School of Medical Sciences, University of Bristol, University Walk, Bristol BS8 1TD, UK

**Keywords:** colorectal cancer, ionising radiation, G2 checkpoint, UCN-01

## Abstract

Ionising radiation is commonly used in the treatment of colorectal cancer. Tumour cells with mutant p53 undergo cell cycle arrest at G2/M after ionising radiation and evidence suggests that abrogation of this G2 arrest can lead to a premature, aberrant mitosis, thus enhancing ionising radiation-induced cell killing. The G2 checkpoint inhibitor UCN-01 was thus investigated to determine whether it would abrogate the G2 checkpoint induced by 5 Gy ionising radiation in a range of colorectal tumour cell lines. Data presented show that, at doses that are alone non-toxic to the cells, UCN-01 inhibits the ionising radiation-induced G2 checkpoint in five colorectal tumour cell lines with mutant p53. The ability of UCN-01 to sensitise cells to ionising radiation-induced growth inhibition and apoptosis was also investigated and UCN-01 was found to radiosensitise two out of five cell lines. These results were confirmed by long-term colony forming efficiency studies. These results demonstrate that abrogation of the ionising radiation-induced G2 checkpoint is not necessarily associated with sensitisation to ionising radiation, however, some colorectal tumour cell lines can be radiosensitised by UCN-01. Although the mechanism of radiosensitisation is not clear, this may still be an important treatment strategy.

*British Journal of Cancer* (2002) **87**, 352–358. doi:10.1038/sj.bjc.6600492 www.bjcancer.com

© 2002 Cancer Research UK

## 

Mutations in the p53 tumour suppressor gene have been observed in many epithelial tumours and leukaemias (Hollstein *et al*, 1991), including approximately 80% of colorectal carcinomas (Vogelstein *et al*, 1988), and this can be associated with resistance to IR-induced cell death in some cell types (Lee and Bernstein, 1993; Nagasawa *et al*, 1994; Siles *et al*, 1996). After DNA damage, for example by ionising radiation (IR), cells undergo arrest at the G1 and G2 phases of the cell cycle. The G1 arrest is dependent on wild type p53 function (Kastan *et al*, 1991; Kuerbitz *et al*, 1992), whereas the G2 arrest can occur in the absence of wild type p53 (Kastan *et al*, 1991; Bracey *et al*, 1995), however p53 may play a role in the G2 arrest in some cell types (Agarwal *et al*, 1995; Hermeking *et al*, 1997). Cell cycle checkpoints are believed to allow time for DNA repair and therefore, in tumour cells with mutated p53, the G2 checkpoint is the last remaining point at which DNA damage can be repaired prior to mitosis and cell division (reviewed in Weinert, 1998).

Abrogation of the G2 checkpoint has been associated with sensitisation of tumour cells to DNA damaging agents. For example, agents such as the methylxanthines caffeine and pentoxifylline have been shown to abrogate the G2 checkpoint and increase sensitivity to IR, presumably due to the ability of these agents to cause premature, and hence lethal, mitosis after IR (Powell *et al*, 1995; Bracey *et al*, 1997). Abrogation of the G2 checkpoint and subsequent sensitisation to DNA damage-induced cell death is suggested to be specific to cells that do not express wild type p53, as these cells depend on the G2 arrest for survival after DNA damage (Powell *et al*, 1995; Bunch and Eastman, 1996; Wang *et al*, 1996; Bracey *et al*, 1997). Hence this may be an important treatment strategy as tumour cells may be preferentially targeted whilst causing minimal disruption to surrounding non malignant tissue. It has previously been demonstrated that colorectal tumour cell lines are radiosensitised by caffeine in a mutant p53-specific manner (Bracey *et al*, 1997), however caffeine is inappropriate for use in humans as doses required to achieve this effect show central nervous system and cardiac toxicities.

7-hydroxystaurosporine (UCN-01) was originally isolated from a *Streptomyces* species as an inhibitor of protein kinase C (PKC) (Akinaga *et al*, 1991; Mizuno *et al*, 1993). UCN-01 has anti-tumour properties alone (Akinaga *et al*, 1991; Seynaeve *et al*, 1994) and has also been shown to abrogate the G2 checkpoint and to sensitise tumour cells to various DNA damaging agents (Bunch and Eastman, 1996; Wang *et al*, 1996; Yu *et al*, 1998). In view of these properties, UCN-01 has recently undergone phase I clinical trials (Senderowicz and Sausville, 2000). Potential targets of UCN-01 are currently under much investigation and it is suggested to act specifically at the G2/M transition. Cell cycle arrest at G2 is tightly controlled by a series of phosphorylation reactions (reviewed in Hwang and Muschel, 1998; Russell, 1998). In short, IR induces a G2 arrest through inactivation of the cyclin B/Cdc2 complex. Cyclin B/Cdc2 is retained in an inactive state by phosphorylation at threonine 14 and tyrosine 15, and dephosphorylation of these residues by Cdc25C is critical for activation and progression to mitosis (reviewed in Zhou and Elledge, 2000). UCN-01 has been shown to inhibit Chk 1 which phosphorylates and inactivates Cdc25C, and UCN-01 is proposed to cause premature entry into mitosis in this way (Busby *et al*, 2000; Graves *et al*, 2000).

Colorectal cancer is the second most common cause of cancer death in the Western world and hence improvement of existing treatments will significantly reduce the morbidity and mortality associated with this disease. Previous work by Wang *et al* (1996) has shown that UCN-01 sensitises the colorectal carcinoma cell line HT29 to IR-induced growth inhibition, however, it is not known whether this is a general response of all colorectal tumour cells. Given the frequent use of radiotherapy in treatment of colorectal cancer and the potential importance of this therapeutic strategy, the aim of this study was to determine whether UCN-01 was able to abrogate the IR-induced G2 arrest in a range of colorectal tumour cell lines, and whether this would consistently lead to radiosensitisation in these cell lines.

## MATERIALS AND METHODS

### Cell lines and culture conditions

HT29 is derived from a human sporadic colon carcinoma (Fogh and Trempe, 1975) and has mutant p53 (Rodrigues *et al*, 1990). SW480 is derived from a human rectal carcinoma (Leibovitz *et al*, 1976) and has mutant p53; SW620 was derived from a lymph node when this cancer recurred with widespread metastasis (Leibovitz *et al*, 1976). S/KS is derived from a human sporadic rectal carcinoma (Hague *et al*, 1993) and expresses high levels of truncated p53 (Williams *et al*, 1993). The S/RG/C2 cell line is derived from a human sporadic colorectal adenoma and is hemizygous at the p53 locus, and the remaining allele is mutated (Baker *et al*, 1990).

The carcinoma (HT29, SW480, SW620 and S/KS) and adenoma (S/RG/C2) cell lines were maintained in Dulbecco's Modified Eagle's Medium (DMEM) supplemented as described previously (Bracey *et al*, 1995, 1997). All cell lines were grown in 25 cm^2^ (T25) tissue culture flasks and maintained at 37°C in 5% CO_2_ LEEC dry incubators.

### Treatment with 5* *Gy IR*±*UCN-01

Cells were seeded in duplicate flasks in standard growth medium at a density of either 1×10^6^ (carcinomas) or 2×10^6^ (adenoma S/RG/C2). Cells in the exponential phase of growth were exposed to 5 Gy γ IR using a ^137^Cs generator (Gravatom) at a dose rate of 0.33 Gy per min (as described previously, Bracey *et al*, 1995). Control cultures were mock irradiated. Immediately after IR cells were treated with normal growth medium containing either 25, 50 or 300 nM UCN-01 (Drug Synthesis and Chemistry Branch, Developmental Therapeutics program, Division of Cancer Treatment, National Cancer Institute) which was prepared as a 10 mM stock solution in DMSO. Cells were grown for 7 days and the medium was replaced with fresh medium containing UCN-01 every 3–4 days, or with fresh medium after 24 h, depending on experiment.

### Assessment of cell cycle distribution

Cell cycle arrest after IR was determined using a FACScan (Becton Dickinson), as described previously (O'Connor *et al*, 1993; Bracey *et al*, 1995). Either 12, 18 or 24 h after IR, duplicate flasks of cells were trypsinised and cells were stained with propidium iodide prior to analysis on a FACScan using CellQuest software (distributed by Becton Dickinson). Cell cycle distribution was determined using either Modfit (Verity Software House, ME, USA) or WinMDI (Joseph Trotter, Scripps Institute) software.

### Mitotic index

The percentage of mitotic cells after IR and/or UCN-01 was determined to confirm that cells underwent a ‘true’ G2 cell cycle arrest that was completely abrogated. Cells were seeded onto glass slides at a density of 0.5×10^6^ cells per slide. After 3 days, slides were irradiated with 5 Gy and/or UCN-01 or mock irradiated. At various times after IR cells were fixed and stained with the DNA dye Hoechst 33258 (0.5 μg ml^−1^; Sigma). Mitotic cells were scored for the characteristics of mitosis, that is visible, evenly stained chromosomes as described previously (Bunz *et al*, 1998), and at least 400 cells were scored for each sample.

### Treatment with colcemid

The mitotic inhibitor colcemid (Gibco) was added to exponentially growing cells (grown on slides as described above) immediately after IR and/or UCN-01 treatment at a dose of 0.01–0.04 μg ml^−1^ depending on cell line. Preliminary experiments were carried out to determine the appropriate concentration of colcemid (HT29, 0.04 μg ml^−1^; SW620, 0.01 μg ml^−1^; S/RG/C2, 0.02 μg ml^−1^). At specific timepoints after treatment mitotic index was determined as described above.

### Determination of attached cell yield

Attached cell yield was calculated in order to determine cell survival after treatment with IR and/or UCN-01. Duplicate flasks were trypsinised and attached cells were quantified using a counting chamber.

### Measurement of apoptosis

We have previously shown that 5 Gy IR can induce apoptosis in colorectal adenoma and carcinoma cell lines (Bracey *et al*, 1995, 1997). Cells that are detached from the monolayer and float into the culture medium show both morphological and biochemical features of apoptosis and the majority of cells that remain attached do not show features of apoptosis (Hague *et al*, 1993; Bracey *et al*, 1995; Shureiqi *et al*, 2000). The medium was removed from the flasks every 3-4 days and numbers of cells with characteristics of apoptosis were counted. Apoptosis was confirmed by acridine orange staining (as described previously, Hague *et al*, 1993, Bracey *et al*, 1995); floating cells were stained with 5g/ml acridine orange, and analysed by fluorescent microscopy for morphological features of apoptosis, most obviously condensed chromatin (as described in Hague *et al*, 1993). At least 200 cells were scored for each treatment. The proportion of floating cells that were apoptotic did not significantly vary between treated and untreated cells and therefore the number of floating cells was used to indicate % apoptosis, which was calculated as number of floating cells as a % of the total cell number. (described previously, Hague *et al*, 1993; Bracey *et al*, 1995).

The attached cell population was also analysed for the presence of apoptotic cells by acridine orange staining; in all cell cultures less than 1% of the attached population showed morphological features of apoptosis. The attached population was also examined for the formation of giant, polyploid cells. These are cells that are proposed to arise from checkpoint disruption and the completion of multiple rounds of DNA synthesis without mitosis (Waldman *et al*, 1996), leading to cell death. Attached cells were stained with acridine orange and cells of greater than 30 μm diameter were scored as giant, using an eye piece graticule. In this investigation control cells were less than 25 μm diameter (described previously, Bracey *et al*, 1997). In all cell cultures there was no evidence of an increase in giant cells in the attached population.

### Colony forming efficiency

After 7 days of treatment with IR and/or UCN-01 attached cell yield was determined (as described above) and 1×10^3^ cells from each treatment were then re-seeded in T25 flasks with fresh (untreated) medium. The flasks were incubated for a further 14 days and medium was replaced every 3–4 days during this time. The attached cells were then fixed in 10% formaldehyde (in PBS; BDH) overnight and then stained with methylene blue (Sigma) overnight. The methylene blue dye was then washed off with water and colonies of greater than 50 cells were counted and colony forming efficiency (CFE) was calculated as a proportion of the original cell number seeded.

### Statistical analysis

A two-tailed, paired, students *t*-test was performed on combined treatments of IR and UCN-01, to determine whether there was a significant difference between treatment with a combination of UCN-01 and IR, compared to IR alone. A *P* value of <0.05 was taken as significant and *=*P*<0.05, **=*P*<0.01, ***=*P*<0.001.

## RESULTS

### UCN-01 abrogates the G2 arrest induced by 5 Gy IR

Colorectal tumour cell lines with mutant p53 treated with 5 Gy IR arrest at the G2 phase of the cell cycle and this is maximal 12 to 24 h after IR, depending on cell line (Bracey *et al*, 1995). To determine the effect of UCN-01 on the G2 cell cycle checkpoint, four colorectal carcinoma-derived cell lines and one colorectal adenoma-derived cell line were irradiated with 5 Gy and then incubated in medium containing UCN-01 for 12–24 h, depending on cell line, and then prepared for analysis by FACS.

Preliminary studies were carried out to determine a dose of UCN-01 that alone was non-toxic to the cell lines over a 7 day period. As shown in Table 1

**Table 1:**
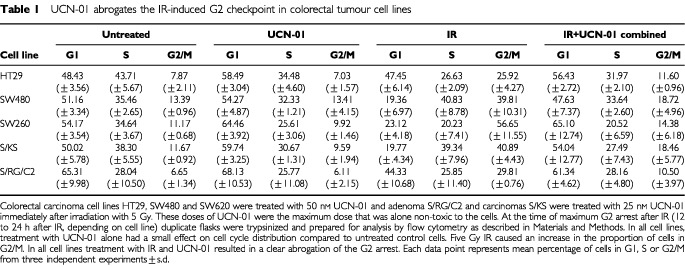
UCN-01 abrogates the IR-induced G2 checkpoint in colorectal tumour cell lines

, treatment with non-toxic doses of UCN-01 (25–50 nM) alone had very little effect on cell cycle distribution. The colorectal carcinoma cell lines HT29, SW480, SW620, S/KS and the adenoma cell line S/RG/C2 all undergo a delay at the G2 phase of the cell cycle after 5 Gy IR.

Treatment with IR and UCN-01 resulted in a clear abrogation of the G2 arrest (Table 1). In all five cell lines, IR causes an accumulation of cells in the G2 phase of the cell cycle and addition of UCN-01 results in a reduction of cells in G2, similar to control untreated levels. In all cell lines the cell cycle arrest at G2/M was confirmed to be a true G2 arrest by determination of the mitotic index (as described in Materials and Methods). For example, in the HT29 cell line, the percentage of mitotic cells in unirradiated cultures remained constant (5.3±1.13%). In the cultures treated with 5 Gy IR, mitotic frequency was decreased to <1% 12 h after IR and remained low (<2%) at 24 h post IR. By 48 h post IR the mitotic frequency was similar in treated and control cell cultures (see also Table 2

**Table 2:**
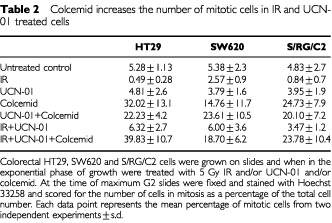
Colcemid increases the number of mitotic cells in IR and UCN-01 treated cells

for S/RG/C2 and SW620 data). To confirm that UCN-01 was having an effect at the G2 checkpoint and not prior to the checkpoint, experiments were carried out using colcemid which is known to inhibit spindle formation and therefore blocks M phase. As expected colcemid treatment alone resulted in an increase in the number of cells in mitosis in HT29, SW620 and S/RG/C2 (Table 2). IR and UCN-01 combined treatment had little effect on the mitotic index consistent with UCN-01 inhibiting the IR induced G2 arrest. Furthermore, IR and UCN-01 in the presence of colcemid resulted in a large increase in the mitotic index in all three cell lines (Table 2) showing that the cells are indeed progressing through G2 then being inhibited in M phase by the colcemid. These data are consistent with UCN-01 having its effect at the G2 checkpoint and thus resulting in IR and UCN-01-treated cells progressing through M phase.

### Radiosensitisation by UCN-01 is seen in two colorectal carcinoma cell lines

We next examined whether the combined treatment of IR and UCN-01 results in a decrease in cell yield (compared to either treatment alone) and/or an increase in the proportion of apoptotic cells (compared to either treatment alone). Apoptosis was examined in the floating and attached cell populations.

**Figure 1:**
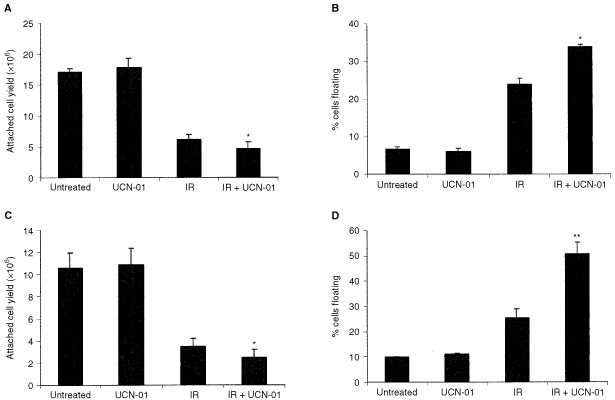
Radiosensitisation of carcinomas HT29 and SW480 by UCN-01. Survival of the HT29 (**A**) and SW480 (**C**) carcinoma cell lines, which have mutant p53, was assessed by attached cell yield after 7 days treatment with 5 Gy IR and/or 50 nM UCN-01. Treatment with UCN-01 alone had no effect on the attached cell yield compared to untreated control cells. IR alone reduced cell yield and this was further significantly reduced by combined treatment with IR and UCN-01. Data represents means of three independent experiments±s.d.; *Significant reduction in cell yield in IR and UCN-01 (combined) cultures compared to IR alone, *P*<0.05. Induction of apoptosis of the HT29 (**B**) and SW480 (**D**) carcinoma cell lines was assessed by determining the percentage of cells which had detached from the monolayer and were floating, these cells were then determined to be apoptotic as described previously (Bracey *et al*, 1995), also see Materials and Methods. Treatment with UCN-01 alone had no effect on levels of apoptosis. After treatment with both 5 Gy IR and 50 nM UCN-01 (combined) there was a significant increase in apoptosis compared to IR alone. Data represents means of three independent experiments±s.d.; *Significant increase in floating cells in IR and UCN-01 (combined) cells compared to IR alone, *=*P*<0.05, **=*P*<0.01.

Attached cell yield was used as a determinant of cell survival 7 days after IR. In all cell lines investigated 5 Gy IR caused a reduction in cell yield (for example, Figures 1a,c

and 2a

**Figure 2:**
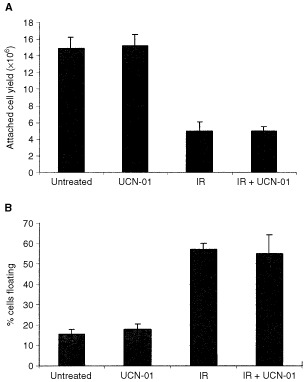
UCN-01 did not radiosensitise adenoma S/RG/C2. (**A**) Attached cell yield was determined as a measure of cell survival 7 days after 5 Gy IR and/or treatment with 25 nM UCN-01. Treatment with 25 nM UCN-01 alone had no effect on attached cell yield compared to untreated controls. IR alone reduced cell yield and this was not further increased by IR in combination with UCN-01. Data represents the mean of three independent experiments±s.d. (**B**) Induction of apoptosis was assessed by the percentage of floating cells as described previously (Bracey *et al*, 1995) and in Materials and Methods. Treatment with UCN-01 alone had no effect on levels of apoptosis. Five Gy IR induced apoptosis but this was not further significantly increased by IR in the presence of UCN-01. There was no evidence of an increase in apoptosis in the attached population in cells treated with IR and UCN-01 compared to IR alone, nor was there any evidence of cell death via formation of giant polyploid cells – see Results.

). Addition of UCN-01 after IR resulted in a further significant reduction in cell yield compared to IR alone in the HT29 and SW480 cell lines (*P*<0.05, Figure 1a,c). In the S/RG/C2, SW620 and S/KS cell lines addition of UCN-01 did not result in any additional reduction in cell yield when compared to IR alone (for example S/RG/C2, Figure 2a).

It was also important to determine whether the observed reduction in cell yield was due to an increase in apoptosis. As described previously, the level of apoptosis in cultured epithelial cell lines can be assessed by measuring the proportion of cells that have detached from the monolayer and are floating in the medium (Hague *et al*, 1993; Bracey *et al*, 1995). These cells are then examined for morphological features of apoptosis, most obviously condensed chromatin. In both HT29 and SW480 cell lines the reduction in cell yield seen in cells treated with a combination of IR and UCN-01 compared to IR alone was associated with a significant increase in the proportion of floating cells that were found to be apoptotic (*P*<0.05 and *P*<0.01, Figure 1b,d). There was no evidence of an increase in apoptosis in S/RG/C2, SW620 and S/KS in cultures treated with a combination of IR and UCN-01 compared to IR alone (for example S/RG/C2, Figure 2b).

Although an increase in apoptosis is usually detected by an increase in the proportion of floating cells, it is possible that the attached cells may show features of apoptosis. Therefore, in all cases the attached cell populations were also examined for characteristics of apoptosis. However, there was no increase in apoptosis in attached cells (for example in S/RG/C2 the level of apoptosis in the attached cell population was consistently less than 1% in control and treated cultures). These cells were also investigated for the presence of giant polyploid cells. These are cells which are proposed to have undergone multiple rounds of DNA synthesis in the absence of mitosis which may result from checkpoint disruption (Waldman *et al*, 1996). We have previously shown that in colorectal cell lines the G2 checkpoint after IR can be abrogated by 1.5 mM caffeine and that in some cell lines radiosensitisation is not associated with an increase in apoptosis but with an increase in giant cells in the attached population (Bracey *et al*, 1997). We examined those cells lines in which checkpoint abrogation was not associated with radiosensitisation, as assessed by increase in apoptosis, for the presence of giant cells in the attached population. There was no increase in giant cells in those cultures treated with IR and UCN-01 combined, compared to IR alone (data not shown).

### A higher dose of UCN-01 does not radiosensitise S/RG/C2

Data shown above demonstrate that the HT29 and SW480 cell lines were radiosensitised by treatment with 50 nM UCN-01 (Table 1 and Figure 1). Although treatment with 25 nM UCN-01 (a non-toxic dose in S/RG/C2) abrogated the IR-induced G2 checkpoint in S/RG/C2 (Table 1), there was no evidence of radiosensitisation in this cell line (Figure 2). It was of interest, therefore, to determine whether a higher dose of UCN-01 (300 nM) for a shorter treatment period (24 h) (a protocol described previously (Wang *et al*, 1996)), would be more effective at abrogation of the G2 checkpoint and would result in sensitisation to IR-induced cell death in the S/RG/C2 cell line which was not previously sensitised. In both the HT29 and S/RG/C2 cell lines, treatment with 300 nM UCN-01 effectively abrogated the IR-induced G2 arrest. HT29 was radiosensitised, similar to that seen with 50 nM treatment for 7 days as described above (data not shown). However, despite using this high dose of UCN-01 and following a previously published protocol (Wang *et al*, 1996), the S/RG/C2 cell line was still not sensitised to IR-induced growth inhibition or apoptosis (data not shown).

### Long-term colony forming efficiency assays confirmed radiosensitisation

**Figure 3:**
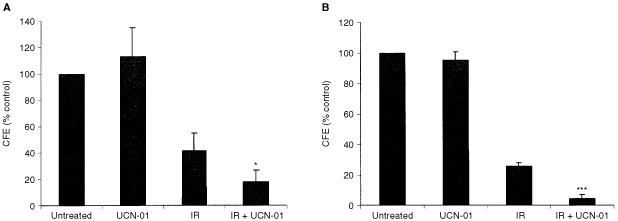
Long-term survival studies of HT29 and SW480. After continuous 7 day treatment with IR and/or UCN-01 colorectal cell lines HT29 and SW480 were trypsinised and a known number of cells was re-seeded into control medium without UCN-01. After a further 14 days of growth cells were fixed, stained and colony forming efficiency (CFE) was calculated (as described in Materials and Methods). (**A**) The CFE of colorectal carcinoma-derived cell line HT29 was not significantly affected by treatment with UCN-01 alone. IR reduced CFE. Cells which had been treated with IR and UCN-01 had a significantly reduced CFE compared to cells treated with IR alone (*P*<0.05). (**B**) The CFE of colorectal carcinoma-derived cell line SW480 was not affected by treatment with UCN-01 alone. IR reduced CFE. Cells which had been treated with IR and UCN-01 had a significantly reduced CFE compared to cells treated with IR alone (*P*<0.001).

The colorectal cell lines HT29, SW480 and S/RG/C2 were further investigated to determine whether UCN-01 had any effect on long-term survival after treatment with 5 Gy IR. After 7 days of treatment with IR and/or a non-toxic dose of UCN-01, cells were trypsinised and re-seeded at a known density in control medium without UCN-01. After a further 14 days of growth, cells were fixed, stained and CFE was calculated. Results presented in Figure 3a,b

show that the CFE of both the HT29 and SW480 cell lines was not affected by UCN-01 alone but that CFE was significantly reduced in those cells treated with a combination of IR and UCN-01 compared to IR alone (*P*<0.05 and *P*<0.001; HT29 and SW480 respectively). Furthermore the CFE of S/RG/C2 was not significantly reduced in those cells treated with IR and UCN-01 combined, compared to IR alone (*P*=0.17, data not shown), thus these long-term studies confirm the observations of 7 day cell survival.

## DISCUSSION

The present study set out to determine whether abrogation of the IR-induced G2 checkpoint by UCN-01 would lead to sensitisation of colorectal tumour cells to 5 Gy IR. Previous work has shown that abrogation of the G2 checkpoint can potentiate cell death induced by a range of DNA damaging agents (Powell *et al*, 1995; Bunch and Eastman, 1996; Wang *et al*, 1996; Bracey *et al*, 1997; Li *et al*, 1998). Using colorectal adenoma and carcinoma-derived cell lines Bracey *et al* (1997) showed that the IR-induced G2 arrest could be overcome by caffeine, leading to radiosensitisation, however caffeine has cardiac and central nervous system toxicities at doses required to inhibit the G2 checkpoint. In the present study we wished to determine whether radiosensitisation could be achieved in a range of colorectal adenoma and carcinoma-derived cell lines (with mutant p53), using UCN-01 at non-toxic doses that can be tolerated by animals and humans (Hill *et al*, 1994; Fuse *et al*, 1998; Sausville *et al*, 1998).

Addition of UCN-01 immediately after 5 Gy IR results in an inhibition of the G2 arrest in all five cell lines investigated. We then investigated whether abrogation of the G2 checkpoint after IR was associated with increased sensitivity to IR. We confirmed the previous report by Wang *et al* (1996) that HT29 can be sensitised to IR by UCN-01, interestingly, however, radiosensitisation was not seen in all cell lines where the G2 arrest was abrogated. In two carcinoma cell lines (HT29 and SW480), abrogation of the G2 arrest lead to radiosensitisation as shown by significantly reduced 7 day cell survival in those cells treated with IR and UCN-01, compared to IR alone. This reduced cell yield was associated with a significant increase in proportion of cells undergoing apoptosis, and this data was confirmed by colony forming efficiency studies which showed that long-term survival of HT29 and SW480 treated with IR and UCN-01 was significantly reduced, compared to IR alone.

Unexpectedly in two out of four carcinoma cell lines (SW620 and S/KS) and in the adenoma cell line (S/RG/C2), abrogation of the G2 arrest after IR did not result in sensitisation to IR. Cell survival and apoptosis were equal in cells treated with IR and UCN-01 compared to cells treated with IR alone. This contrasts with previous data published by our group which has shown that abrogation of the IR-induced G2 checkpoint by caffeine is associated with radiosensitisation in all colorectal cell lines investigated with mutant p53 (Bracey *et al*, 1997). Although radiosensitisation is usually confirmed by analysis of attached and floating (apoptotic) cells, we wished to confirm that there was not an increase in apoptosis in the attached cell populations. An increase in morphological features of apoptosis or an increase in giant polyploid cells in the attached population may have indicated radiosensitisation which would not have been detected by analysis of floating cells. There was no evidence, however, for an increase in apoptosis or giant cell formation in the attached cell population in those cultures treated with combined IR and UCN-01 compared to either treatment alone. The 7 day cell survival and apoptosis data were also confirmed by colony forming efficiency data which indicated that the long-term survival of S/RG/C2 after IR was not further reduced by the presence of UCN-01.

Thus it appears, at least in colorectal tumour cell lines, that inhibition of the IR-induced G2 checkpoint is not necessarily associated with sensitisation to IR. This in contrast to other reports which have shown that UCN-01 is able to sensitise tumour cells to a range of DNA damaging agents, including IR (Akinaga *et al*, 1993; Bunch and Eastman, 1996; Wang *et al*, 1996; Shao *et al*, 1997; Sugiyama *et al*, 2000). There are other reported cases where abrogation of the G2 checkpoint is not always associated with sensitisation to DNA damage-induced apoptosis (Musk, 1991; Ribeiro *et al*, 1999) however in these cases the G2 checkpoint was overcome by caffeine. Caffeine has multiple cellular effects including inhibition of ATM (Blasina *et al*, 1999; Zhou *et al*, 2000) and it is possible that caffeine may radiosensitise cells through checkpoint-independent mechanisms, such as direct inhibition of DNA repair (Asaad *et al*, 2000).

The observation in the present study that some colorectal tumour cell lines cannot be radiosensitised by UCN-01, despite abrogation of the G2 checkpoint, is intriguing. These data suggest, firstly, that lack of both the G1 and G2 arrest in some cell lines with mutant p53 has a no more detrimental effect on the cells than lack of a G1 arrest after IR. Secondly, these data suggest that the G2 checkpoint may be redundant or ineffective in those cells lines where radiosensitisation was not seen. That is, if the DNA repair mechanisms were not functional or impaired in the cell lines where radiosensitisation was not demonstrated, then this would explain why loss of the G2 checkpoint has no adverse effect on these cells. In this scenario, the cells would be unable to adequately repair IR-induced double-strand breaks and therefore would be expected to undergo apoptosis regardless of the delay at G2. The observation by Bunch and Eastman (1996) that UCN-01 can abrogate the cisplatin-induced G2 arrest in CHO cells regardless of DNA repair status, but that sensitisation to cisplatin is only seen in mismatch repair-proficient cells suggests that UCN-01 may work through inhibition of DNA repair pathways (Bunch and Eastman, 1996). Furthermore Jiang and Yang (1999) propose that UCN-01 suppresses nucleotide excision repair of cisplatin-induced lesions.

In summary, this investigation demonstrates that inhibition of the IR-induced G2 arrest is not necessarily synonymous with sensitisation to IR. In two out of five cell lines, however, radiosensitisation was demonstrated and thus treatment with UCN-01 may still be an important strategy for colorectal cancer. To our knowledge this is the first report of this nature in a range of colorectal tumour cell lines and the observation of cell line-specific radiosensitisation by UCN-01 may be clinically valuable.
